# Combination of Cefditoren and *N*-acetyl-l-Cysteine Shows a Synergistic Effect against Multidrug-Resistant Streptococcus pneumoniae Biofilms

**DOI:** 10.1128/spectrum.03415-22

**Published:** 2022-11-29

**Authors:** Mirella Llamosí, Julio Sempere, Pilar Coronel, Mercedes Gimeno, Jose Yuste, Mirian Domenech

**Affiliations:** a Spanish Pneumococcal Reference Laboratory, National Center for Microbiology, Instituto de Salud Carlos III, Madrid, Spain; b CIBER de Enfermedades Respiratorias (CIBERES), Madrid, Spain; c Scientific Department, Meiji Pharma Spain, Madrid, Spain; d Department of Genetics, Physiology, and Microbiology, Faculty of Biology, Complutense University of Madrid, Madrid, Spain; Emory University School of Medicine

**Keywords:** biofilm, pneumococcus, serotype 19A, antioxidants, *N*-acetyl-l-cysteine, cefditoren

## Abstract

Biofilm formation by Streptococcus pneumoniae is associated with colonization of the upper respiratory tract, including the carrier state, and with chronic respiratory infections in patients suffering from chronic obstructive pulmonary disease (COPD). The use of antibiotics alone to treat recalcitrant infections caused by biofilms is insufficient in many cases, requiring novel strategies based on a combination of antibiotics with other agents, including antibodies, enzybiotics, and antioxidants. In this work, we demonstrate that the third-generation oral cephalosporin cefditoren (CDN) and the antioxidant *N*-acetyl-l-cysteine (NAC) are synergistic against pneumococcal biofilms. Additionally, the combination of CDN and NAC resulted in the inhibition of bacterial growth (planktonic and biofilm cells) and destruction of the biofilm biomass. This marked antimicrobial effect was also observed in terms of viability in both inhibition (prevention) and disaggregation (treatment) assays. Moreover, the use of CDN and NAC reduced bacterial adhesion to human lung epithelial cells, confirming that this strategy of combining these two compounds is effective against resistant pneumococcal strains colonizing the lung epithelium. Finally, administration of CDN and NAC in mice suffering acute pneumococcal pneumonia caused by a multidrug-resistant strain was effective in clearing the bacteria from the respiratory tract in comparison to treatment with either compound alone. Overall, these results demonstrate that the combination of oral cephalosporins and antioxidants, such as CDN and NAC, respectively, is a promising strategy against respiratory biofilms caused by S. pneumoniae.

**IMPORTANCE** Streptococcus pneumoniae is one of the deadliest bacterial pathogens, accounting for up to 2 million deaths annually prior to severe acute respiratory syndrome coronavirus 2 (SARS-CoV-2). Vaccines have decreased the burden of diseases produced by S. pneumoniae, but the rise of antibiotic-resistant strains and nonvaccine serotypes is worrisome. Pneumococcal biofilms are associated with chronic respiratory infections, and treatment is challenging, making the search for new antibiofilm therapies a priority as biofilms become resistant to traditional antibiotics. In this work, we used the combination of an antibiotic (CDN) and an antioxidant (NAC) to treat the pneumococcal biofilms of relevant clinical isolates. We demonstrated a synergy between CDN and NAC that inhibited and treated pneumococcal biofilms, impaired pneumococcal adherence to the lung epithelium, and treated pneumonia in a mouse pneumonia model. We propose the widely used cephalosporin CDN and the repurposed drug NAC as a new antibiofilm therapy against S. pneumoniae biofilms, including those formed by antibiotic-resistant clinical isolates.

## INTRODUCTION

According to recent data from the World Health Organization (1), respiratory tract diseases remain one of the main causes of death in the world in 2019. Lower respiratory tract infections are the world’s most deadly communicable disease and are the fourth leading cause of death without considering severe acute respiratory syndrome coronavirus 2 (SARS-CoV-2) pandemic data, whereas chronic obstructive pulmonary disease (COPD) claimed another 3 million lives worldwide in 2019 (1). In addition, acute respiratory infections are the main cause of mortality in children under 5 years, with Streptococcus pneumoniae (pneumococcus) being the principal cause of severe pneumonia in both developing and developed countries. Acquisition of new strains of S. pneumoniae is associated with acute exacerbations of COPD (AECOPD) in current and former smokers (2, 3), with this bacterial pathogen being one of the most frequent in COPD patients, although many other chronic bacterial infections are also produced by communities of diverse species (4).

Notably, 65 to 80% of chronic bacterial infections are caused by microbes growing in biofilms (5). The presence of biofilms is well documented in otitis media and persistent pneumonia, which are difficult diseases to treat with antibiotics and are often chronic and recurrent. Most of the information available so far is related to S. pneumoniae (6). The inherent tolerance of these communities to antibiotic therapy and the host immune system is well known (7–10). Biofilms may be established on medical devices, such as indwelling intravenous catheters or endotracheal tubes, when local defenses are impaired (11). The ability of respiratory pathogens to persist in the nasopharynx and cause infection under the appropriate conditions is associated with the ability to form biofilms on the mucosal epithelium (12). Current strategies for inhibition of biofilm formation or disrupting established biofilms represent an exciting new approach to the treatment of chronic infectious diseases. The application of these strategies to respiratory tract infections also offers a better understanding of the significance of mucosal biofilm in the pathogenesis of these conditions.

S. pneumoniae carriage is a prerequisite to the later invasion of sterile sites, leading to what is known as invasive pneumococcal disease (IPD). Indeed, the pneumococcus is responsible for episodes of sepsis, bacteremic pneumonia, and meningitis, mainly in children, the elderly, and immunocompromised patients (13). In addition, pneumococci are the main bacterial cause of noninvasive diseases such as nonbacteremic pneumonia, acute otitis media, sinusitis, and conjunctivitis (14). One of the major threats in pneumococcal infection is the emergence of nonvaccine serotypes, causing IPD associated with antibiotic resistance (15, 16).

Numerous strategies are being actively pursued to inhibit biofilm formation or eradicate established biofilms of respiratory pathogens (17, 18). Bacteria forming a biofilm can be up to 1,000 times more resistant to antimicrobial agents than planktonic cells (19), and in this sense, new antibiofilm therapies are necessary from the prophylactic and therapeutic perspectives as alternative or complementary options to common antibiotics. The mucolytic agent *N*-acetyl-l-cysteine (NAC), a precursor of the antioxidant glutathione, has been investigated for its antimicrobial activity in the inhibition of biofilm formation and in the clearance of developed biofilms. The ability of NAC to interfere with bacterial growth and biofilm formation was first demonstrated against Staphylococcus epidermidis, confirming a concentration-dependent effect (20). Since then, many other studies have demonstrated the efficacy of NAC in reducing biofilm formation in a wide range of microorganisms (including Gram-negative and Gram-positive bacteria, as well as yeasts), demonstrating its ability to impair matrix architecture and promote the disruption of biofilms (6, 21–23). In addition, the combination of antibiotics with other alternative therapies is a promising and effective strategy against bacterial biofilms (24, 25).

In this study, we used cefditoren (CDN), a third-generation oral cephalosporin, which has demonstrated bactericidal activity against many Gram-negative and Gram-positive bacterial pathogens. Previous publications have shown that CDN has good *in vitro* activity against the two major bacterial respiratory pathogens, S. pneumoniae and Haemophilus influenzae (26). The main objective of this work was to explore the ability of cefditoren alone and in combination with NAC to inhibit (prevent) and degrade (treat) biofilms produced by S. pneumoniae serotype 19A strains with different antibiotic susceptibility patterns.

## RESULTS

### Cefditoren showed a marked antimicrobial activity against S. pneumoniae (planktonic and biofilm).

**FIG 1:**
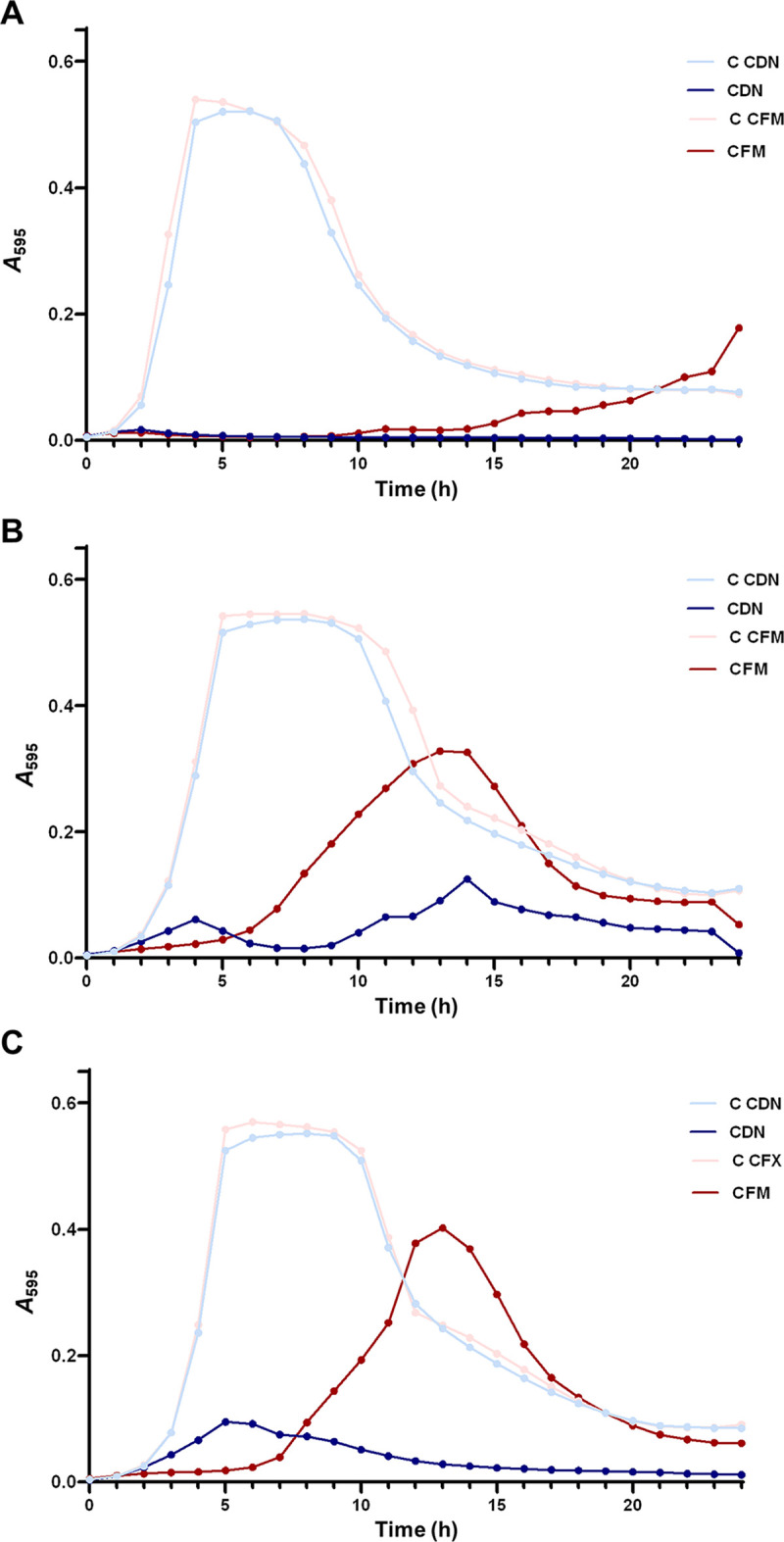
Inhibition of S. pneumoniae growth curves with third-generation cephalosporins. CDN (0.4 μg/mL) or CFM (0.4 μg/mL) were added at the beginning with the inoculum, and the cultures were incubated for 24 h at 37°C. (A) Growth curve of the susceptible strain 3064/19. (B) Growth curve of the intermediate strain 1171/19. (C) Growth curve of the MDR strain 3152/19. C, control.

To explore the ability of CDN to prevent S. pneumoniae growth and biofilm formation, we used clinical strains of serotype 19A with different susceptibilities to CDN (3064/19, susceptible; 1171/19, intermediate; and 3152/19, resistant). To compare the results obtained with CDN, we used the antibiotic cefixime (CFM), which is also a third-generation oral cephalosporin. First, we tested the bacteriolytic activity of CDN and CFM separately against the bacterial growth of the different clinical isolates (Fig. 1A to C). The results clearly proved that CDN displays a strong bacteriolytic effect, even against the multidrug-resistant (MDR) strain 3152/19 (Fig. 1C), whereas when CFM was used, the viability of the culture was recovered after 8 h of incubation with the antibiotic, even for the susceptible strain, 3064/19 (Fig. 1A). These results confirm that CDN has stronger antimicrobial activity than CFM.

**FIG 2:**
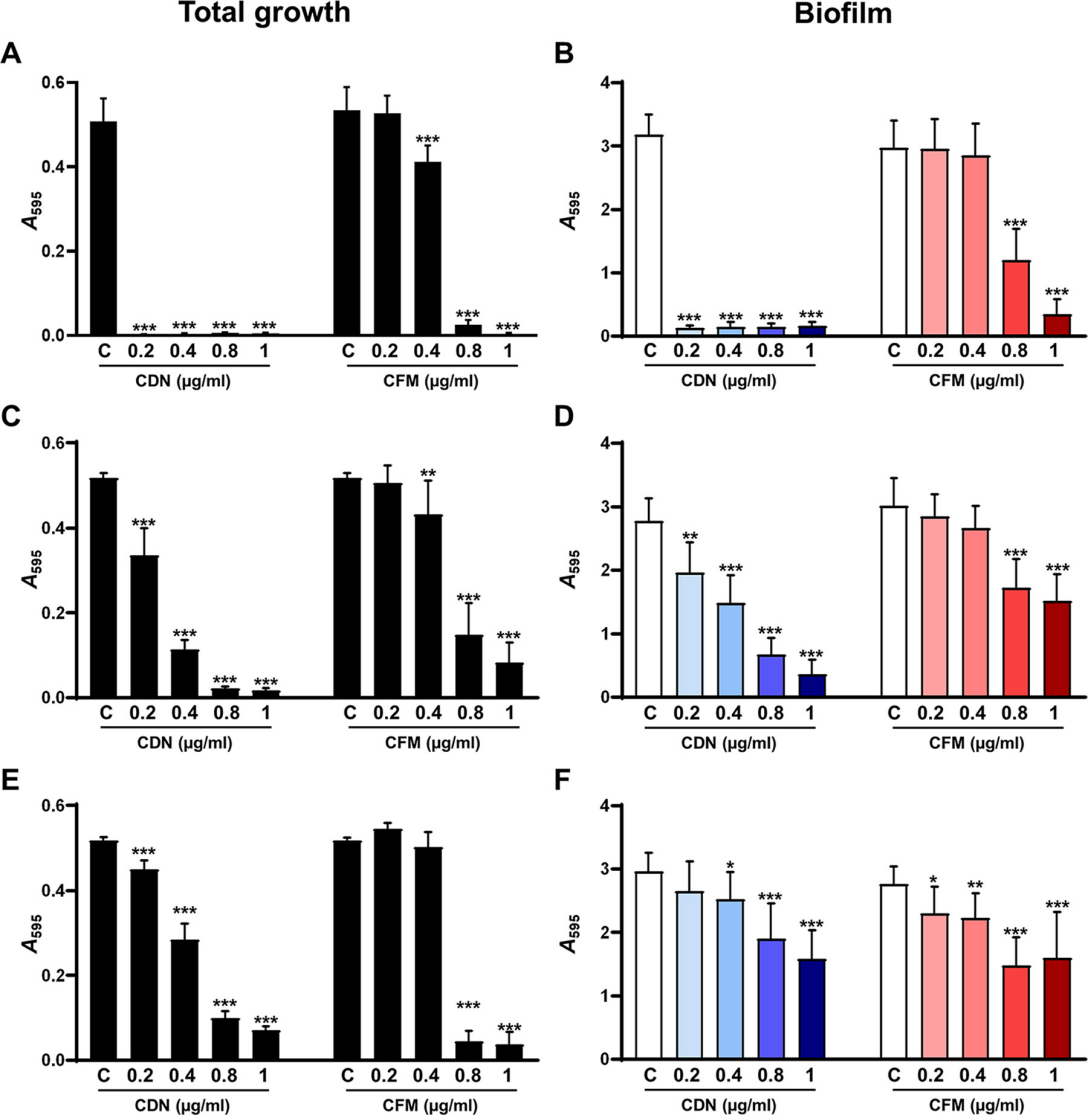
Inhibition (prevention) of S. pneumoniae biofilms with the third-generation cephalosporins CDN and CFM. Different concentrations of CFM or CDN (0 to 1 μg/mL) were added at the beginning with the inoculum, and the biofilms were incubated for 5 h at 37°C. We used the susceptible strain 3064/19 (A and B), the intermediate strain 1171/19 (C and D), and the MDR strain 3152/19 (E and F). Black bars represent the total growth (planktonic plus biofilm) (A, C, and E), and the white/colored bars represent the biofilm biomass (B, D, and F). Data represent the average of three experiments. Standard deviation bars are shown, and asterisks mark results that are statistically significant (two-tailed Student’s *t* test: *, *P* < 0.05; **, *P* < 0.01; ***, *P* < 0.001).

In addition, we evaluated the effect of different concentrations of CDN and CFM against bacterial growth (including planktonic and biofilm) and against established biofilms (biofilm biomass) of the different S. pneumoniae strains growing in microwell plates (Fig. 2). We performed an inhibition (prevention) assay, where different concentrations of CDN or CFM (0.2, 0.4, 0.8, and 1 μg/mL) were added at the beginning of the incubation along with the inoculum (Fig. 2). For this purpose, we measured the total growth and biofilm biomass after 5 h of incubation (Fig. 2). Treatment with CDN was very effective at inhibiting the total bacterial growth for all the clinical isolates (Fig. 2A, C, and E). For the susceptible strain (3064/19), exposure to the lowest dose of CDN (0.2 μg/mL) was enough to show an almost complete eradication of pneumococcal cells, whereas for CFM, concentrations up to four times higher were necessary to observe a similar effect (0.8 μg/mL) (Fig. 2A). For the intermediate (1171/19) and resistant (3152/19) strains, higher doses of CFM than CDN were required to reduce the total growth of the pneumococcal cells (Fig. 2C and E). In terms of prevention of pneumococcal biofilm biomass (Fig. 2B, D, and F), an almost complete reduction in the biofilm of the susceptible strain (3064/19) was observed with the lowest concentration of CDN (0.2 μg/mL), producing a 96% reduction of the pneumococcal biofilm (Fig. 2B). However, for CFM, a concentration up to 5 times higher (1 μg/mL) was necessary to achieve a similar reduction of the pneumococcal biofilm (Fig. 2B). In the pneumococcal strain with intermediate resistance to CDN (1171/19), the use of CDN as a preventive measure showed effectiveness in inhibiting the biofilm formation at all the doses evaluated, leading to a 90% reduction of the biofilm with a dose of 1 μg/mL (Fig. 2D). However, for CFM, higher doses were necessary to show a certain degree of reduction, confirming that CFM had a weaker ability to prevent biofilms by nonsusceptible strains than CDN (Fig. 2D). In the case of the pneumococcal strain resistant to CDN and CFM, the inhibitory effect of both antibiotics against the pneumococcal biofilm was similar, showing bactericidal activity with increasing doses (Fig. 2F).

### Effects of the combination of CDN and NAC on the growth, formation, and disaggregation of S. pneumoniae biofilms.

**FIG 3:**
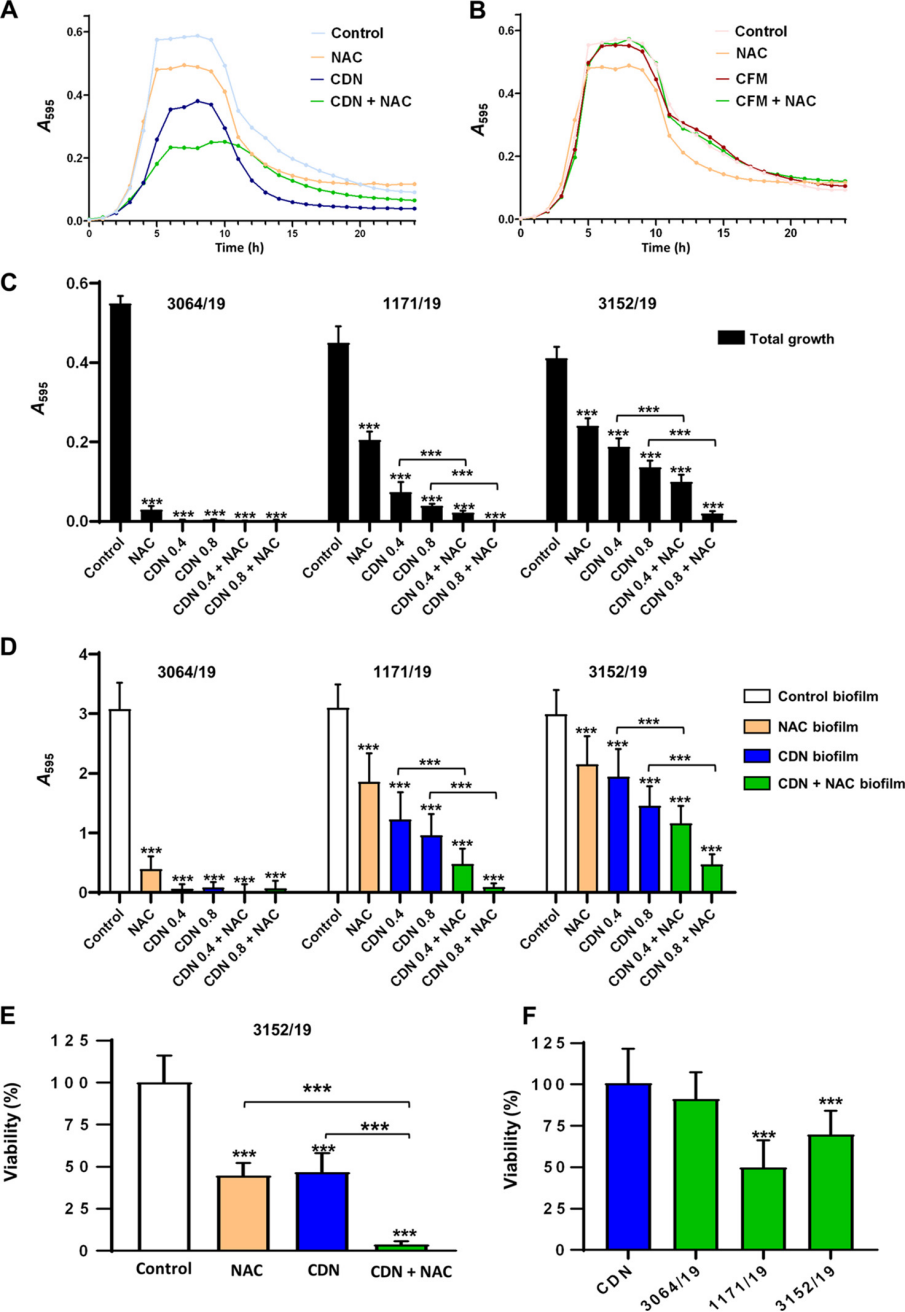
Effect of the combination of NAC and CDN/CFM on the growth, formation, and disaggregation of S. pneumoniae biofilms. (A and B) Effect of treatments on growth curves of the MDR strain S. pneumoniae 3152/19. NAC (1.5 mg/mL), CDN (0.4 μg/mL), CFM (0.4 μg/mL), or a combination of antibiotic plus antioxidant was added at the beginning with the inoculum, and the cultures were incubated for 24 h at 37°C. (C and D) Inhibition of S. pneumoniae biofilms with different concentrations of NAC (2.5 mg/mL), CDN (0.4 or 0.8 μg/mL), or a combination of NAC and CDN. Treatments were added at the beginning with the inoculum, and the biofilms were incubated for 5 h at 37°C. We used the susceptible strain 3064/19, the intermediate strain 1171/19 and the MDR strain 3152/19. (E) Viable counts showing the inhibition (prevention) of MDR strain 3152/19 biofilm with CDN and NAC. NAC (2.5 mg/mL), CDN (0.8 μg/mL), or a combination of NAC and CDN was added at the beginning with the inoculum, and the biofilms were incubated for 5 h at 37°C. Viability was assessed at the end of the experiment and normalized to the control. (F) Viable counts showing the disaggregation (treatment) of S. pneumoniae biofilms with CDN and NAC. Biofilms were inoculated and incubated for 4 h at 37°C and then treated with CDN (2 μg/mL) or a combination of NAC (2.5 mg/mL) and CDN for 1.5 h at 37°C. Viability was assessed at the end of the experiment and normalized to CDN. Data represent the average of three experiments. Standard deviation bars are shown, and asterisks mark results that are statistically significant (two-tailed Student’s *t* test: ***, *P <* 0.001).

We characterized the combined effect of cephalosporins and NAC against pneumococcal biofilms of the different strains of S. pneumoniae (Fig. 3). First, we evaluated the impact of adding NAC, CDN, and CFM alone and a combination of NAC with each cephalosporin on the growth curves of the MDR strain 3152/19 (Fig. 3A and B). Our results confirmed that the combination of 1.5 mg/mL of NAC and 0.4 μg/mL of CDN was more effective than individual treatments at inhibiting the growth of this resistant pneumococcal strain (Fig. 3A). However, the combination of NAC with CFM at the same concentrations had no effect (Fig. 3B). Thereafter, we only tested NAC and CDN.

We also analyzed the combined effect of NAC and CDN on the total growth and biofilm of the different serotype 19A strains in a prevention/inhibition assay (Fig. 3C and D). Treatment with CDN was more effective than treatment with NAC at preventing the formation of pneumococcal biofilms for all the investigated strains (Fig. 3C and D). For the susceptible strain (3064/19), individual treatments with 2.5 mg/mL of NAC or 0.4 to 0.8 μg/mL of CDN caused a dramatic decrease in the growth, and therefore, additive or synergistic effects mediated by the combination of NAC with CDN were not possible to distinguish from individual treatments (Fig. 3C). However, for the intermediate (1171/19) and resistant strains (3152/19), the combination of NAC and CDN achieved greater effectiveness in reducing the total growth (Fig. 3C).

In terms of inhibition of the biofilm biomass (Fig. 3D), for the susceptible strain (3064/19), the individual treatments were highly effective, as we observed for the total growth, and we could not quantify the impact of the combination (Fig. 3D). However, for the intermediate and resistant strains of serotype 19A, much higher antimicrobial activity was observed when NAC and CDN were combined in comparison to the individual treatments, confirming the potential effectiveness of this combination in the prevention of pneumococcal biofilms (Fig. 3D). Based on these results, further studies exploring the activity against pneumococcal biofilms were performed using the resistant strain (3152/19) (Fig. 3E). Viability studies demonstrated that NAC and CDN had a similar effect against pneumococcal cells within the biofilm, yielding the observation that the combination of 2.5 mg/mL of NAC and 0.8 μg/mL of CDN reduced the biofilm viability by up to 95%, indicating a stronger effect compared to the individual treatments with CDN or NAC (Fig. 3E). Overall, these results confirm a potential synergistic effect between NAC and CDN on the growth and formation of pneumococcal biofilms.

To set up the optimal conditions for treatment assays with established pneumococcal biofilms, different amounts of CDN (0.5 to 6 μg/mL) were tested to evaluate the ability of CDN to disaggregate established mature pneumococcal biofilms. For the MDR strain (3152/19), the viability of the biofilm was reduced by 1 log at 0.5 μg/mL and up to 2 logs at 2 μg/mL (data not shown). Based on these preliminary results, a combination of 2.5 mg/mL of NAC with 2 μg/mL of CDN was used for the disaggregation assays against the three different pneumococcal strains of serotype 19A (Fig. 3F). The combined treatment resulted in a 9% reduction in the CDN-susceptible strain (3064/19), a 50% reduction in the CDN intermediate strain (1171/19), and a 30% reduction in the MDR strain (3052/19) in comparison with treatment with CDN alone (Fig. 3F). Hence, the synergistic effect between NAC and CDN was demonstrated for all the strains with reduced susceptibility to CDN.

### Treatment with the combination of CDN and NAC impaired the attachment of S. pneumoniae to the lung epithelium and was effective against pneumococcal pneumonia.

**FIG 4:**
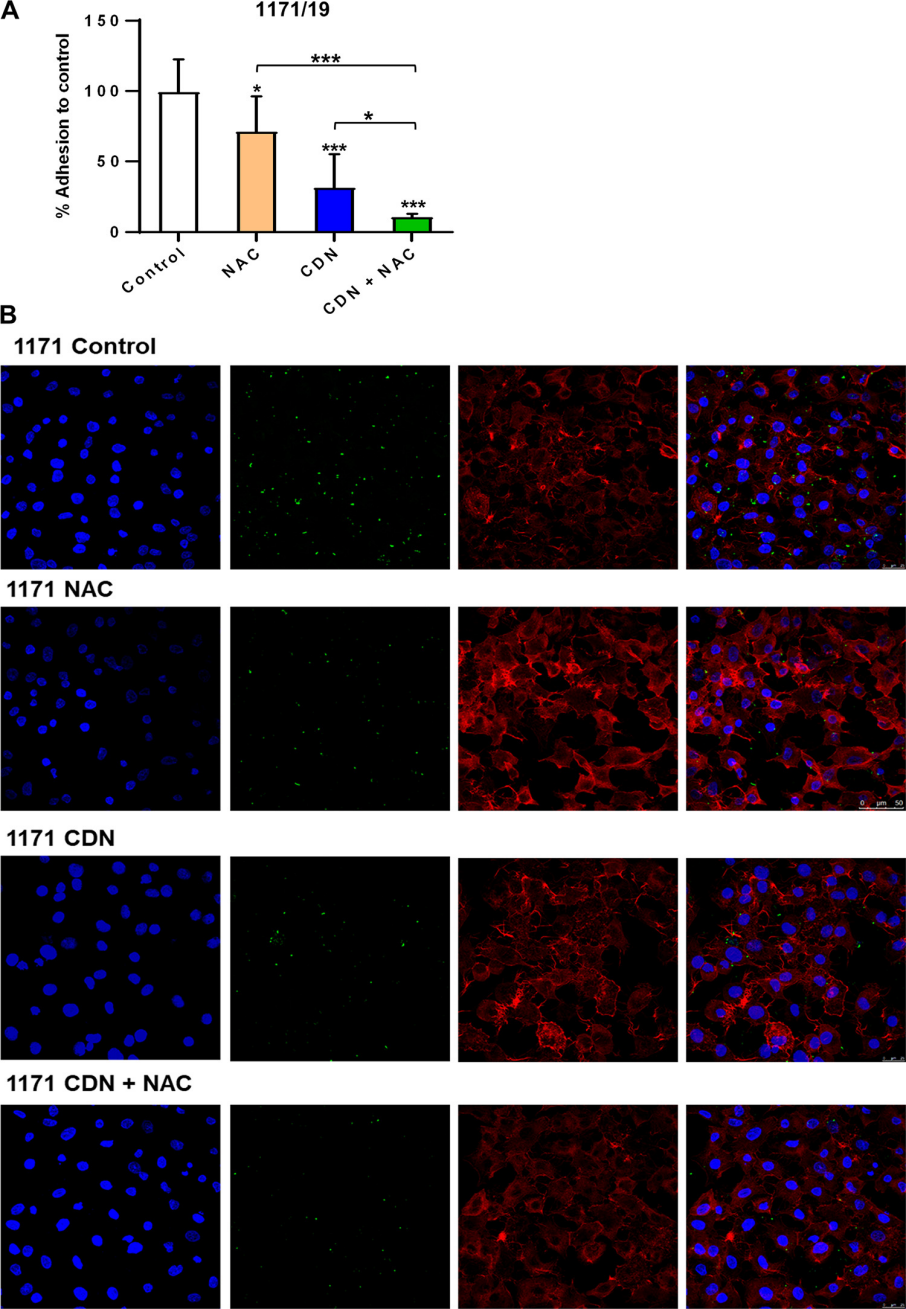
Effect of the combination of NAC and CDN on adhesion of the intermediate strain S. pneumoniae 1171/19 to the lung epithelium. A549 cells were prepared to a density of 10^5^ cells per well, infected with strain 1171/19, and incubated for 2 h at 37°C. The culture was then treated with NAC (2.5 mg/mL), CDN (0.4 μg/mL), or a combination of CDN plus NAC and incubated for 1 h at 37°C. (A) Box and whisker plot showing viability. Viability was assessed at the end of the experiment and normalized to the control. Data represent the average of three experiments. Standard deviation bars are shown, and asterisks mark results that are statistically significant (two-tailed Student’s *t* test: *, *P < *0.05; ***, *P < *0.001). (B) DNA was stained with Hoechst solution, the actin cytoskeleton was visualized with rhodamine-phalloidin staining, and bacterial strain 1171/19 was fluorescently labeled with FAM-SE. Images represent the *xy* from z-stacks at 0.5-μm intervals.

**FIG 5:**
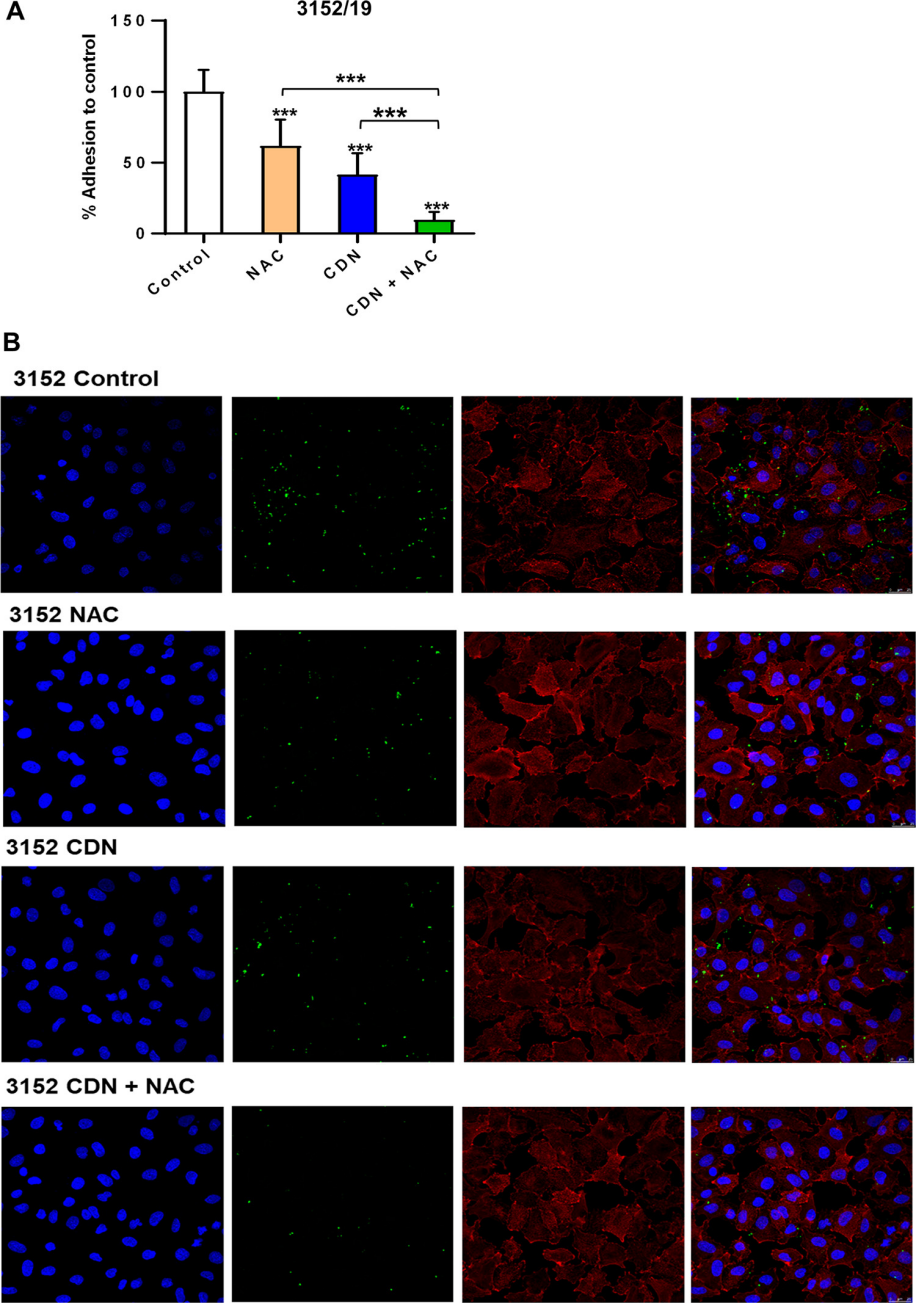
Effect of the combination of NAC and CDN on adhesion of the MDR strain S. pneumoniae 3152/19 to the lung epithelium. A549 cells were prepared to a density of 10^5^ cells per well, infected with strain 3152/19, and incubated for 2 h at 37°C. The culture was then treated with NAC (2.5 mg/mL), CDN (0.4 μg/mL), or a combination of CDN plus NAC and incubated for 1 h at 37°C. (A) Box and whisker plot showing viability. Bars graph showing viability was assessed at the end of the experiment and normalized to the control. Data represent the average of three experiments. Standard deviation bars are shown, and asterisks mark results that are statistically significant (two-tailed Student’s *t* test: *, *P < *0.05; ***, *P < *0.001). (B) DNA was stained with Hoechst solution, the actin cytoskeleton was visualized with rhodamine-phalloidin staining, and bacterial strain 3152/19 was fluorescently labeled with FAM-SE. Images represent the *xy* from z-stacks at 0.5-μm intervals.

The antimicrobial effect of the combination of CDN and NAC against pneumococcal adhesion in the lower respiratory tract was explored using human lung epithelial cells. We studied the individual effect of both drugs and the combination by analyzing the viable bacterial counts, and images of the infected cells were obtained using confocal laser scanning microscopy (CLSM). For these assays, pneumococcal strains with reduced susceptibility to CDN (strains 1171/19 and 3152/19) were used (Fig. 4 and 5). In the viability assay, the percentage of adhesion compared to the control was determined, and we found that exposure to 0.4 μg/mL of CDN was more effective than 2.5 mg/mL of NAC at reducing the adhesion of both S. pneumoniae strains (Fig. 4A and 5A). These results agree with previous findings shown above related to the prevention of biofilm assays (Fig. 3). Moreover, the combination of CDN and NAC reduced the adhesion to a higher degree (*P < *0.001) than each compound individually, demonstrating again a synergistic effect (Fig. 4 and 5). In the CLSM images, we also observed an increased effect of the combined treatment compared to the individual treatments for both strains (Fig. 4B and 5B). These results indicate that the combination of CDN and NAC displays a synergistic antimicrobial effect against pneumococcal attachment to lung epithelial cells by clearing the adhesion of the bacteria to the respiratory tract (Fig. 4B and 5B).

**FIG 6:**
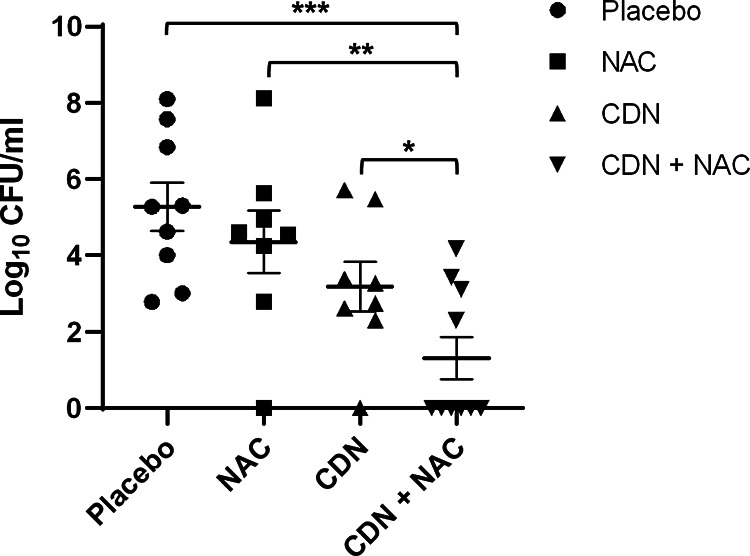
Effect of the combination of NAC and CDN in a mouse pneumonia model. Mice were infected with MDR strain 3152/19 and then treated (1 dose every 12 h) with humanized doses of NAC, CDN, and a combination of CDN plus NAC. Results are expressed as bacterial counts obtained from the lungs. Standard deviation bars are shown, and asterisks mark results that are statistically significant (two-tailed Student’s *t* test: *, *P* < 0.05; **, *P* < 0.01; ***, *P* < 0.001).

To evaluate the *in vivo* relevance of our findings, a pneumonia model of infection using the MDR strain (3152/19) was studied in mice to mimic pneumococcal pneumonia. For this purpose, mice anesthetized with isoflurane were infected via intranasal inoculation with the infective strain resistant to CDN. After 24 h of infection, the mice were treated with 1 dose every 12 h of humanized doses of NAC (5.7 mg/kg), CDN (4.3 mg/kg), or the combination, as recommended by the manufacturer; the mice were sacrificed at 48 h postinfection to analyze the bacterial levels in the lungs. In Fig. 6, we show the bacterial counts (log_10_ CFU per milliliter) recovered from the lungs of infected mice at 48 h postinfection (after 24 h of treatment). We did not see significant differences in the groups treated with NAC or CDN individually (Fig. 6), which is consistent with the adhesion assays using human lung epithelial cells. However, treatment with the combination of CDN and NAC significantly reduced the bacterial load in the lung compared to the individual treatments, confirming that the CDN and NAC combination is synergistic against pneumococcal pneumonia, even against a strain resistant to CDN (Fig. 6).

## DISCUSSION

One of the main challenges at present is how to treat bacterial biofilms, when antibiotic therapy alone is futile, especially if the biofilms are formed by antibiotic-resistant strains. Moreover, the use of antibiotic therapy alone can complicate the outcome of the infection process, as it imposes a selective pressure that can end with the emergence of new antibiotic-resistant strains (25, 27). In the search for antimicrobial alternatives to overcome these recalcitrant infections, several authors have suggested combining antibiotics with antibodies (24), antioxidants (25), or even phage lysins (28). In the case of respiratory infections, patients suffering from COPD have recurrent pneumonia episodes that, in many cases, are caused by the same strains that are colonizing their lower respiratory tract (29, 30). In these COPD patients, S. pneumoniae is one of the major etiologic agents causing acute exacerbations (29, 30). Antibiotics are widely used in these patients, and therefore, the risk of developing antibiotic resistance is an additional complication (29, 30). In the current study, when we compared the activity of CDN and CFM, both third-generation oral cephalosporins that are frequently used against respiratory infections, we observed that CDN was more effective than CFM. CDN showed a greater inhibition of the total growth and biofilm formation of S. pneumoniae. This is consistent with recent reports showing that CDN had higher activity against S. pneumoniae clinical isolates, with lower MIC_50_ and MIC_90_ values than other third-generation cephalosporins, including CFM and cefpodoxime (15, 16).

In this study, as a complementary treatment against pneumococcal biofilms, we used the mucolytic agent NAC, which presents thiol-reactive groups (31). This compound not only has antimicrobial properties but also can be used to treat a wide range of respiratory diseases, such as COPD and asthma (32). Moreover, the antioxidant and anti-inflammatory properties of this compound have been used to treat cytokine storm and respiratory distress in coronavirus disease 2019 (COVID-19) patients (31). The antimicrobial properties of NAC could be attributed to the competence of cysteine uptake by bacteria, based on the disulfide bond reduction of proteins (affecting bacterial adhesion and biofilm formation), or perturbation of the redox intracellular equilibrium (33, 34). Additionally, it has been suggested that NAC could act as a weak acid in the biofilm, acidifying the biofilm matrix and bacterial cytoplasm (35). In this sense, we recently demonstrated that NAC showed antimicrobial activity against polymicrobial biofilms of S. pneumoniae and S. aureus that were susceptible and resistant to methicillin (36).

We examined the efficacy of administration of the third-generation oral cephalosporins CDN and CFM and the combination of each with NAC for the treatment and prevention of pneumococcal biofilms. Treatment with CDN in combination with NAC reduced the growth and biofilm formation of different strains of S. pneumoniae to a much higher degree than the mixture of CFM with NAC or treatment with the cephalosporins individually. This is consistent with previous studies showing that NAC has an excellent safety and efficacy profile when used as an adjuvant molecule in the treatment of bacterial biofilms (33, 37–39). The administration of NAC in association with tigecycline has been suggested as an alternative therapy to treat catheter-associated infections (i.e., urinary tract infections, device-related infections) (40). Moreover, in combination with different antibiotics (vancomycin, rifampicin, ciprofloxacin, azithromycin, and tobramycin), NAC significantly promoted inhibition and/or eradication of bacterial biofilms (41–43). One aspect that reinforces the relevance of our findings is that few studies to date have focused on the combined treatment of biofilm-associated respiratory tract infections. In particular, the effect of NAC and ciprofloxacin on 15 different strains of Pseudomonas aeruginosa isolated from patients with suppurative otitis media (44) confirmed the lack of growth when NAC (5 mg/mL) was used for treatment, either alone or in combination with ciprofloxacin (44). Another study using 11 S. pneumoniae strains showed that NAC alone had little activity against the planktonic and sessile cultures, but when it was combined with three different antibiotics (amoxicillin, erythromycin, and levofloxacin), a slightly enhanced activity against biofilms was observed in some of the strains (45).

One aspect that reinforces the relevance of our findings is that few studies to date have focused on the combined treatment of biofilm-associated respiratory tract infections. Moreover, there are no previous reports in the literature studying the combination of NAC with oral cephalosporins to prevent and treat S. pneumoniae biofilms affecting the respiratory tract. In this sense, we have demonstrated the synergistic effect of CDN and NAC to reduce S. pneumoniae adhesion to the lung epithelium using A549 cells and for the treatment of acute pneumonia in a mouse model of infection. Hence, our results are the first studies demonstrating the synergistic effect between oral cephalosporins and antioxidants (here, CDN and NAC) for avoiding pneumococcal adhesion in the lower respiratory tract and treating pneumococcal pneumonia. The *in vivo* antimicrobial activity of both compounds was established using an MDR serotype 19A clinical isolate, as this serotype is prevalent in relapses and reinfections in COPD patients associated with biofilms of the lower respiratory tract (46, 47). This is largely relevant for patients suffering from chronic respiratory infections such as COPD, who can develop recurrent and persistent infections by S. pneumoniae and other respiratory pathogens (29, 30). One of the main problems with COPD patients is the lack of complete clearance of the infection from the lower respiratory tract (48, 49), causing recurrent infections. Our findings promote the potential of CDN and NAC not only to reduce S. pneumoniae adhesion to the lung epithelium but also to treat acute pneumonia episodes in which biofilms are already established. The use of NAC as an adjunctive therapy to treat S. pneumoniae meningitis in combination with ceftriaxone has been tested in a mouse meningitis model, although they did not find any therapeutic benefit (50). However, our findings suggest that the combination of CDN and NAC could be useful for treating respiratory infections associated with biofilms, as we found a significant reduction in pneumococcal biofilms, including prevention of their formation and clearance once established, and a decrease in the bacterial load in the lung compared to the individual treatments.

Overall, our study demonstrates that the combination of CDN with NAC can prevent and treat S. pneumoniae biofilms and reduce the adhesion of S. pneumoniae to the lung epithelium, thus representing a promising treatment against pneumococcal pneumonia. These results encourage a more widespread clinical use of NAC as an adjuvant to different antibiotics, such as CDN, especially in microbial infections followed by biofilm settlement (pneumonia, COPD exacerbations, otitis media, sinusitis), therefore increasing the potential application of the combination of CDN and NAC as a preventive and therapeutic treatment for microorganisms producing bacterial biofilms.

## MATERIALS AND METHODS

### Strains, media, and culture conditions.

Table 1 shows the pneumococcal strains examined in this study. We used clinical isolates of S. pneumoniae from our collection at the Spanish Pneumococcal Reference Laboratory (SPRL) (National Center for Microbiology, Instituto de Salud Carlos III [ISCIII]). All strains were from serotype 19A because it is a prevalent serotype frequently associated with multidrug resistance, including to β-lactams, and because biofilm formation is markedly high in this particular serotype (12, 51). The pneumococcal strains were cultured on Mueller-Hinton blood agar plates and incubated at 37°C and 5% CO_2_. Moreover, for liquid medium culture, all strains were grown in CpH8 medium supplemented with 0.08% yeast extract (C+Y medium). Growth was controlled by measuring the optical density at 550 nm (OD_550_).

**TABLE 1:** Strains used in this study and MIC values for the different antibiotics and NAC^*a*^

Strain	Serotype (source, description)	Source	MIC (μg/mL)									MIC (mg/mL)
			PEN	AMX	CFM	CDN	CTX	TET	CHL	ERY	LVX	NAC
3064/19	19A (pediatric/blood; bacteremic pneumonia)	SPLR	0.12	0.12	2	0.12	0.25	0.5	4	0.25	1	2.5
1171/19	19A (pediatric/cerebrospinal fluid; meningitis)	SPLR	2	2	>16	0.5	1	32	2	>128	1	2.5
3152/19	19A (pediatric/blood; bacteremia without focus)	SPLR	4	8	>16	1	4	32	4	>128	1	2.5

a SPRL, Spanish Pneumococcal Reference Laboratory; PEN, penicillin; AMX, amoxicillin; CFM, cefixime; CDN, cefditoren; CTX, cefotaxime; TET, tetracycline; CHL, chloramphenicol; ERY, erythromycin; LVX, levofloxacin; NAC, *N*-acetyl-l-cysteine.

### Susceptibility testing and growth curves.

The susceptibility of the different S. pneumoniae strains of serotype 19A growing as planktonic cultures to the different β-lactams and the antioxidant NAC was determined using the broth microdilution method, following the latest CLSI guidelines (52). The MICs of penicillin (PEN), amoxicillin (AMX), CFM, CDN, and other antibiotics for the different strains are shown in Table 1. The MIC of the antioxidant NAC is also shown in Table 1. CDN (oral cephalosporin) does not have breakpoints following the EUCAST criteria. The strains in this study would be susceptible or susceptible with increased exposure (intermediate) using the breakpoints for cefotaxime (parenteral cephalosporin) or susceptible, intermediate, and resistant if the breakpoints for cefpodoxime (oral cephalosporin) were considered.

For the activity assays on growing cells (growth curves), pneumococcal cells were grown into an early stationary-phase culture in C+Y medium (OD_550_ ≈ 0.5 to 0.6) and then diluted 100-fold into fresh C+Y medium. Aliquots (200 μL per well) (4.5 × 10^6^ CFU/mL) were added to a 96-well polystyrene microtiter plate (Falcon 3095; Corning). Different concentrations of CFM, CDN, and NAC and combinations of an antibiotic plus the antioxidant were added at the beginning and then plates were incubated at 37°C in an Epoch 2 device (BioTek Instruments), which acted as an incubator and read the OD_550_ value every half hour.

### Biofilm formation assay and antibiofilm therapy.

Biofilm formation by pneumococcal cells of serotype 19A was induced using treated 96-well polystyrene microtiter plates (Costar 3595; Corning) as previously described (53). Briefly, cells were grown in C+Y medium up to the early stationary phase (OD_550_ ≈ 0.5 to 0.6), sedimented by centrifugation, resuspended in an equal volume of prewarmed C+Y medium, diluted 100-fold, and then dispensed at a concentration of 200 μL per well (4.5 × 10^6^ CFU/mL).

For the prevention/inhibition assays, different concentrations of CDN and NAC and combinations of the two were added to the bacteria at the start of incubation on the plates, followed by 5 h of incubation at 37°C. Analysis of the effect was explored by crystal violet (CV) staining and viable cell counting, as previously described (6, 10). For CV staining, after the 5-h incubation, total growth (*A*_595_) was measured using the BioTek Epoch 2 reader and then CV (0.2%) was added to stain the biofilm, which was then incubated at room temperature for 15 min, followed by three washes with distilled water. After solubilization with 95% ethanol, the biofilm biomass (*A*_595_) was quantified in the reader. For viability (CFU per milliliter), after the 5-h incubation, the planktonic culture was separated, washed with phosphate-buffered saline (PBS), and gently disaggregated using a pipette. Tenfold serial dilutions were prepared in PBS and then plated in the mentioned blood agar plates and incubated overnight at 37°C and 5% CO_2_. Viable cells were quantified and expressed as CFU per milliliter.

For the treatment/disaggregation assays, different concentrations of CDN and NAC alone and combined were added to the preformed biofilm after 4 h of incubation at 37°C. Analysis of the effect was explored by viable cell counting. Briefly, after incubating the biofilm for 4 h at 37°C, the planktonic culture was separated, and the biofilm was washed with PBS. Then, the different concentrations of antibiotics and antioxidants were added, and the biofilms were incubated for 1.5 h at 37°C, rinsed again with PBS, and gently disaggregated using a pipette. Tenfold serial dilutions were then prepared in PBS. Viable cells were quantified and expressed as CFU per milliliter.

### Adhesion assays and confocal laser scanning microscopy.

Experiments studying adhesion to human epithelial cells were performed using A549 lung cells (CCL-185; ATCC), as previously described (28). Monolayers of cells were cultured to 90 to 95% confluence in tissue culture flasks in RPMI 1640 medium supplemented with 1% HEPES. We used 24-well treated plates (Falcon 353047; Corning) with 10^5^ cells per well that were infected with ≈2 × 10^6^ CFU/mL of strain 1171/19 or 3152/19, achieving a multiplicity of infection (MOI) of 10:1. Then, cells were incubated for 1 h at 37°C. After the initial incubation, the supernatant was separated, the cells were washed three times with PBS, and CDN, NAC, or a combination was added; the cells were incubated for two additional hours at 37°C. After the final incubation, the supernatant was separated, and the cells were washed again with PBS. To lift the infected A549 cells, we first used a solution containing 0.25% trypsin-1 mM EDTA and then 0.025% saponin (in PBS), as previously described (28). From the mixture, 10-fold serial dilutions were prepared in PBS and plated onto blood agar plates. The viable bacterial cells were quantified and expressed as CFU per milliliter.

For the CLSM assays, S. pneumoniae strains 1171/19 and 3152/19 were fluorescently labeled with 6-carboxyfluorescein-succinimidyl ester (FAM-SE; Molecular Probes), as previously described (54, 55). A549 cells infected with FAM-SE-labeled bacteria were added to 12-mm circular coverslips for immunofluorescence staining. We used a staining solution for 30 min at room temperature containing Hoechst solution (Invitrogen) diluted 1/2,500 for DNA staining and rhodamine-phalloidin (Invitrogen) diluted 1/200 for actin cytoskeleton detection. We mounted the samples with Aqua-Poly/mount (Polysciences) and observed them with the Leica spectral SP5 confocal microscope. The images represent the *xy* from z-stacks at 0.5-μm intervals; the images were analyzed using the LAS AF software.

### Pneumococcal pneumonia animal model.

Experimental procedures involving mice were performed at Instituto de Salud Carlos III (ISCIII), conforming to the Spanish government legislation (RD 53/2013, ECC/566/2015) and European Community regulations (2010/63/EU). We used BALB/c male mice (8 to 12 weeks old) weighing about 20 g that were bred by Charles River Laboratories. All animal procedures followed the guidelines of the Bioethical and Animal Welfare Committee of ISCIII and Regional Authorities, which reviewed and approved the protocols (PROEX 063.1/21). To study the treatment of pneumococcal pneumonia with CDN, NAC, or combinations, we used groups of five mice infected as previously described (55), and we performed two independent experiments. To establish the animal model of pneumonia infection, we chose the MDR strain 3152/19 (serotype 19A). Pneumococcal strains harboring antibiotic resistance, such as those in serogroups 9, 14, 19, and 23, generally have low virulence in mice, and therefore, a high dose has to be used (56, 57). Briefly, while the mice were under anesthesia with isoflurane, we inoculated them intranasally with 50 μL of inoculum at a concentration of 10^9^ CFU/mL (5 × 10^6^ CFU per mouse); 24 h after the challenge, the mice were treated with a placebo (PBS) or with humanized doses of CDN (5.7 mg/kg), NAC (4.3 mg/kg), or the combination. We considered the standard human dose of 400 mg/12 h for CDN and 600 mg/day equivalent to 300 mg/12 h for NAC. Treatment was administered twice a day (every 12 h), following the manufacturer’s indications. Finally, 48 h after the challenge, the mice were sacrificed by CO_2_, and bacterial counts were determined from samples recovered from the lung. The results were expressed as CFU per milliliter of bacteria recovered from the lung.

### Statistical analysis.

Data presented in the manuscript represent results obtained from at least three repeated independent experiments, representing at least three replicates. Statistical analyses were performed using GraphPad InStat v. 8.0 (GraphPad Software). For comparison of two groups, we used the two-tailed Student’s *t* test; for multiple comparisons, we used the analysis of variance (ANOVA) test, followed by a *post hoc* test. Differences were considered statistically significant at *P < *0.05 and highly significant at *P < *0.01 and *P < *0.001.
